# Prediction and analysis of functional RNA structures within the integrative genomics viewer

**DOI:** 10.1093/nargab/lqab127

**Published:** 2022-01-14

**Authors:** Warren B Rouse, Ryan J Andrews, Nicholas J Booher, Jibo Wang, Michael E Woodman, Ernst R Dow, Theodore C Jessop, Walter N Moss

## Abstract

In recent years, interest in RNA secondary structure has exploded due to its implications in almost all biological functions and its newly appreciated capacity as a therapeutic agent/target. This surge of interest has driven the development and adaptation of many computational and biochemical methods to discover novel, functional structures across the genome/transcriptome. To further enhance efforts to study RNA secondary structure, we have integrated the functional secondary structure prediction tool ScanFold, into IGV. This allows users to directly perform structure predictions and visualize results—in conjunction with probing data and other annotations—in one program. We illustrate the utility of this new tool by mapping the secondary structural landscape of the human *MYC* precursor mRNA. We leverage the power of vast ‘omics’ resources by comparing individually predicted structures with published data including: biochemical structure probing, RNA binding proteins, microRNA binding sites, RNA modifications, single nucleotide polymorphisms, and others that allow functional inferences to be made and aid in the discovery of potential drug targets. This new tool offers the RNA community an easy to use tool to find, analyze, and characterize RNA secondary structures in the context of all available data, in order to find those worthy of further analyses.

## INTRODUCTION

The roles of RNA secondary structure in mediating key biological functions can be seen across all of life and in every type of RNA (1). The best characterized and most extensively studied structures are found in classical noncoding RNAs (ncRNAs), such as tRNAs, snoRNAs, rRNAs, etc; however, roles of secondary structure in mRNAs and long noncoding RNAs (lncRNAs) are increasingly being appreciated (2–4). Secondary structures are present in many mRNAs where they have been shown to act as recognition sites for RNA binding proteins (RBPs), allow or prevent access of trans-regulatory elements to single stranded RNA, directly bind microRNAs and RBPs, and act to stabilize the transcript (5,6). These secondary structures are also important to every step of RNA processing (7). Due to their large size, the structures of mRNAs are challenging to study, but with their role in post-transcriptional regulation and newly appreciated capacity as a therapeutic target, characterization of these structures is of great importance.

There are a variety of different computational methods that can be used to predict functional secondary structures including RNAz (8), EvoFold (9), CMfinder (10) and GraphClust (11). Many of these structural RNA discovery methods rely on a mix of information, including predicted thermodynamic stability and structural conservation—requiring multiple sequence alignments. These alignments can offer a strong line of support for functionality of a structure if compensatory mutations are present (i.e., evolutionary preservation of specific base pairing) (12); however, this can also limit predictions due to the need for high quality alignment data that has enough variation and evolutionary depth to be informative, restricting the use of these prediction tools to elements that fall within loosely defined ranges of conservation (10,13–14). These limitations drove the development of the functional RNA secondary structure prediction algorithm ScanFold (15).

ScanFold is a two-stage pipeline that decouples the analysis of RNA secondary structure from conservation analysis, allowing a single sequence to be used for identification of potentially functional secondary structures (13,15). In the first stage, ScanFold-Scan, a sliding prediction window is used to find the minimum free energy (MFE) structure of the native sequence using the ViennaRNA package's RNAfold program (16). The native sequence is then randomized (maintaining the same sequence composition) a user defined number of times and the MFEs are recalculated. Using the MFE and partition function values for the native and randomized sequences, ScanFold then calculates several metrics that provide information on thermodynamic stability, accessibility, and propensity to form defined structure (13,15). The window then slides by a user defined number of nucleotides before repeating the process. In the second stage, ScanFold-Fold, the results of all the scanning windows are condensed into a single structural map that is comprised of consensus base pairs across all windows that are consistently more stable than expected (compared to randomized sequence values)—indicating potential functional roles (13,15). These models represent initial structural hypotheses that can be tested against biochemical probing data and phylogenetic comparative data. For example, in our analysis pipeline (illustrated in REF (17)), we pass ScanFold-Fold model secondary structures to the cm-builder program (18) that combines the INFERNAL package (19) and R-Scape (20) to identify potential homologous sequences/structures and evaluate the statistical significance of potential covariation, respectively. In this way, the strengths of ScanFold can be leveraged (unique high-value models that map to specific sequences of interest) to facilitate downstream evaluation using gold-standard phylogenetic approaches.

Of the metrics calculated in the first stage of ScanFold, the thermodynamic *z*-score and ensemble diversity (ED) are the most valuable for structural predictions. The z-score predicts propensity of the sequence to have ordered/evolved secondary structural stability: measured by the number of standard deviations more or less stable (negative or positive *z*-scores respectively) than random the native sequence/structure is, and indicates a potential role of evolution in ordering the sequence to have stable (likely functional) secondary structure (21,22). The ED can also be informative of function as a lower ED is representative of a secondary structure that forms one or a few dominant conformations (22,23), which may be an evolved characteristic of some functional RNAs (24). Additionally, base pair ‘tracks’ (data visualizable on genome browsers) are generated (25). The arcs in these tracks are a color-coded representation of the ScanFold-Fold results, depicting a consensus structure that is formed from recurring base pairs across low *z*-score analysis windows with the greatest bias toward ordered stability and likely functionality (13).

ScanFold is capable of accurately predicting and thermodynamically characterizing functional RNA secondary structures (13,26–30). However, our understanding of RNA biology is rapidly expanding through the use of various large-scale ‘omics’ datasets. For example: biochemical RNA structure probing data can be used to help determine the conformations of mixed populations of RNA (i.e. dynamic RNA structures) (31,32) whereas other annotations can be used to obtain a fuller understanding of a structure's function through locating *cis*- and *trans*-regulatory elements, RBP and microRNA binding sites, and clinically relevant single nucleotide polymorphisms (SNPs). This combination of data has been used to successfully discover functional, and targetable structural motifs (via small molecules) in human mRNAs, such as those for *CNBP, AGO1* and *MBNL1* (30) and recently, in SARS-CoV-2 (33).

ScanFold is a powerful tool for highlighting potentially functional RNA secondary structures, and its original form (available at https://github.com/moss-lab/ScanFold) was catered towards experienced computational biologists. The webserver version (available at https://mosslabtools.bb.iastate.edu/scanfold), makes running ScanFold much easier (13,34) however, many output files must be downloaded, unzipped, converted to genomic coordinates and loaded into a genome browser for visualization and analysis. To circumvent any problems associated with this process and improve the efficiency of finding potentially functional and druggable RNA secondary structures, we integrate ScanFold directly into the IGV graphical user interface (GUI) to both run and visualize ScanFold results locally, alongside complementary data that aids in RNA structure/function analyses.

The current IGV platform is an open-source, cross-platform desktop application that supports browsing of genomic data (35,36) and utilizes base pair visualizations (25). IGV serves as the perfect platform for integration of ScanFold because of its speed, ease of use, ability to compare multiple data sets and file types at once, and its current wide use among researchers: e.g. the original paper describing IGV (35) has been cited over 5000 times. We have created an all-in-one tool for predicting, visualizing, and analyzing large amounts of data for structural characterization of any RNA of interest, which is further enhanced by combining information from the Moss Lab RNAStructuromeDB (37), curated annotation datasets, and biochemical probing data. The use of ScanFold and IGV for studying both human RNAs and viral genomes has proven to be an effective method for functional RNA discovery in our lab and we expect that integration as IGV-ScanFold will have broad utility among members of the RNA community.

To demonstrate the power and utility of IGV-ScanFold, we analyzed the pre-mRNA of a previously studied mature mRNA, *MYC*, in which we had shown the presence of functional secondary structure in the 3′ UTR. Using IGV-ScanFold and all acquired datasets, we were able find and analyze potentially functional secondary structures more comprehensively and efficiently. Our results quickly recapitulated previous data and offered new insights into the functional nature of a promising motif in the 3′ UTR of *MYC*.

## MATERIALS AND METHODS

### Incorporation of ScanFold into IGV

The full code used to incorporate ScanFold into IGV can be found at https://github.com/ResearchIT/scanfoldigv. Several additions to IGV were required, including the generation of a dialog window for choosing parameters and launching ScanFold scripts, and a custom track loader for opening files with a ‘.scanfoldvarna’ extension as VARNA popups. Changes to existing IGV code (https://github.com/ResearchIT/scanfoldigv/blob/master/scanfoldmenu.patch) allows users to run RNAfold and RNAstructure as stand-alone folding algorithms or use either as the folding algorithm in ScanFold. This can be done via a dialog window by (i) clicking on the ‘Region of Interest’ panel or (ii) selecting the new ScanFold menu option. After selecting parameters in the ScanFold dialog window, a python script is run (https://github.com/ResearchIT/scanfoldigv/blob/master/scripts/run_scanfold.py) that receives user settings from IGV as parameters and launches the ScanFold python scripts using those parameters in a platform-specific way. After ScanFold runs, the script generates an IGV batch script in the output directory (https://software.broadinstitute.org/software/igv/batch). This batch script directs IGV to load the output files produced by ScanFold using the standard track loaders built into IGV for each file type. A VARNA 2D model of the predicted structures is created and a new pop-up window with that model is generated. Finally, each build of IGV-ScanFold is packaged for download or sharing using GitHub Actions for Mac (version 10.15 and above) and Windows.

### Annotation acquisition and filtering

All annotation were obtained from publicly available datasets. RefSeq Functional Elements data was downloaded from NCBI (https://www.ncbi.nlm.nih.gov/refseq/functionalelements/) (38). Single nucleotide polymorphism data was downloaded from SNPedia (https://www.snpedia.com/index.php/SNPedia) (39), and NCBI ClinVar database (https://www.ncbi.nlm.nih.gov/clinvar/) (40). MicroRNA data was downloaded from TargetScan (http://www.targetscan.org/vert_72/) (41), and miRBase (http://www.mirbase.org/) (42). ORegAnno data was downloaded from the ORegAnno database (http://www.oreganno.org/) (43). Repeat element data was downloaded from Dfam (https://dfam.org/home) (44). RNA modification data was downloaded from RMBase (http://rna.sysu.edu.cn/rmbase/index.php) (45). PolyA site data was downloaded from PolyASite (https://polyasite.unibas.ch/atlas) (46). RNA binding protein eCLIP data was downloaded from ENCODE (https://www.encodeproject.org/) (47,48). Biochemical probing data was downloaded from the RASP database (http://rasp.zhanglab.net/) (49). The eCLIP data was filtered to remove all low scoring hits (only hits with a score of 1000 were kept), and the ORegAnno data was filtered to remove transcription factors (leaving only RNA relevant data). All data was acquired in the human hg38 genomic coordinate space or, where necessary, was converted via the LiftOver tool (https://genome.ucsc.edu/cgi-bin/hgLiftOver) (50). These curated datasets can be accessed in Data S1.

### Annotation and probing track generation

Due to potential memory limitations, it can be desirable to work with smaller subsets of complementary data (e.g. for a gene of interest). To generate annotation tracks for *MYC*, the FilterBedFiles.py (Python 3.7) script was placed in the annotations folder and used to create gene specific files. This was done using the command ‘$ python FilterBedFiles.py chr#:Genomic Coords OutFileName.bed’ (coordinates and outfile are user defined), and individual annotation tracks were generated. All annotation files were generated in the same way, with the exception of the PolyA bed file. In this case, instead of entering chr# just the chromosome # (e.g. 8 instead of chr8) was used. For the probing datasets, the same approach was used for each type of probing data (in vivo DMS, in vitro DMS, etc.), but after generation of each file a header was added (track type = bedGraph name = ‘Name’ description = ‘Description’) where name and description are user defined. After adding the header to the file, it was searched for the term ‘NA’ and removed to prevent errors when loading into IGV. The use of this script to generate target specific annotations can be seen in Video S3.

### Installing and using IGV-ScanFold

IGV-ScanFold was downloaded, unzipped, and launched using the IGV-ScanFold icon. Once running, the search bar at the top of the window was used to enter the gene name, *MYC*, or coordinates, chr8:127735434–127742951, and navigate to the sequence. ScanFold was then run to find structures with functional propensity. The entire visible region was scanned first by clicking the ‘ScanFold’ tab in the menu bar at the top of the page followed by ‘Run ScanFold’. When the parameters window appeared, the following settings were used: 120 nt window size, 1 nt step size, 100 randomizations per window, mononucleotide shuffling, 37°C, competition of 1, forward strand, and global refold selected. All files were saved to a local directory using the browse button next to the ‘Output Folder’ dialog box and the desired directory was selected. Once ScanFold finished running, all tracks were automatically loaded and aligned to the gene in the IGV window. The resulting tracks were then manipulated based on user preference for ease of viewing and interpretation of the data. A detailed walkthrough of how to start and run IGV-ScanFold can be found in Video S2, and a detailed walkthrough of how to adjust both the view and data displayed on each track can be found in Video S4.

### IGV-ScanFold default limitations

In this implementation of ScanFold the max length sequence allowed to be scanned is 40 kb. If a region larger than this is selected, the Log Output window will simply say ‘Done’ and not complete the scan. Additionally, while completing a scan, no additional scans should be started as this will lead to no output. We have also set the global refold option to be off by default. This prevents any slowdowns or potential for crashes when folding large sequences. If a smaller sequence is being ran, preferably under a few kb, then the global refold option can be selected.

### IGV-ScanFold log outputs and run times

When a scan is started, many things will appear in the Log Output window: for example, what step is being ran ‘ScanFold-Scan running now’, the sequence length being scanned ‘Sequence Length: X nt’, number of windows ‘Approximately X windows will be generated’, and ‘Estimated runtime = X minutes’. The window will update after each step has completed and indicate the current step, actual time to complete the previous step, and the estimated remaining time to completion. These times are estimated based on the number of windows and randomizations needed to complete the scan (i.e. longer sequences, more windows, and more randomizations equates to longer times). These estimated times may be faster or slower depending on the capabilities of the computer being used, therefore, if an older machine with less memory and processing power is being used the run will take much longer than a newer machine with a large amount of memory and processing power.

### Loading data on IGV-ScanFold

Once annotation and probing tracks were generated, they were loaded into IGV alongside all ScanFold tracks. All annotation and probing data were loaded using the menu bar at the top of the screen, File > Load from File and appropriate files were selected. The Phastcons (20-way) conservation track was also loaded using the menu bar at the top of the screen, File > Load File from Server > Annotations arrow > Phastcons (20 way). Other annotations can also be loaded using File > Load from Server or File > Load from URL. A detailed walkthrough of how to load pre-made data tracks and IGV server data tracks can be found in Video S4.

### Generation of 2D models

To make 2D models of the predicted secondary structure, the ExtractedStructures files was used to obtain the correct sequence and structure in dot bracket notation for −2 *z*-score structures. For −1 *z*-score structures, the DBNstructures file (located in the output folder) was used to obtain the correct sequence and structure from the ‘>UserInput_Filter = −1.0’ section in dot bracket notation. To generate a mapping file for biochemical structure probing, data reactivity values for the genomic coordinates of interest were extracted and put into a separate text file. Once the reactivity map file was created, the sequence and structure, in dot bracket notation, were pasted into their respective boxes in the VARNA applet (Version 3.93) and a 2D model was created. Once created, all stems with bulges or loops were straightened manually for clarity of the rendering. The reactivity map was then loaded by right clicking in the main window and under the ‘Display’ tab selecting Color map > Load values. Once loaded and overlayed on the structure, the color map was formatted by right clicking in the main window and under the ‘Display’ tab selecting Color map > Style. The colors associated with reactivity values were changed with reactivities of 0 being white, reactivities of 0.5 being yellow, reactivities of 1.0 being red, and all values between these points being a mixture of those colors. Mapping files for other data types such as average z-score can also be generated in this way using the z-score.wig file. A detailed walkthrough of how to use VARNA to create these models can be found in Video S6.

## RESULTS AND DISCUSSION

### Benefits of using ScanFold integrated in IGV

One benefit of using ScanFold directly in IGV is that it allows users to compare the predicted structures from ScanFold *z*-scores using multiple folding algorithms, e.g. RNAfold (16) and RNAstructure (51). Another major benefit is that additional experimental and annotation data tracks can be loaded, allowing for a comprehensive structural/functional view of the region, which can be used to help determine the best regions for potential drug targeting. The annotation, experimental, and ScanFold file types found to be particularly useful in our work can be found in Table 1, and a list of all IGV-compatible file types can be found on the Broad Institute's website (http://software.broadinstitute.org/software/igv/FileFormats).

**Table 1.** Acceptable IGV file types

File Type	Extension	More Information
Genomic Annotations	GFF, bed, bigBed, wig, bigWig, bedgraph	https://academic.oup.com/bib/article/14/2/178/208453 http://software.broadinstitute.org/software/igv/FileFormats
Chemical Probing	shape, map, bed	http://software.broadinstitute.org/software/igv/FileFormats
Structure (Connectivity Table)	ct	http://software.broadinstitute.org/software/igv/FileFormats
Structure (Dot-Bracket)	db, dbn	http://software.broadinstitute.org/software/igv/FileFormats
Colored Base Pairing	bp	https://academic.oup.com/bib/article/14/2/178/208453
Paring Probabilities	dp	https://academic.oup.com/bib/article/14/2/178/208453
Sequence Alignment	BAM, Goby, maf	http://software.broadinstitute.org/software/igv/FileFormats

Select file types that are accepted by IGV as stated by the Broad Institutes website.

### Obtaining annotation files and biochemical structure probing data

Annotation files and probing data are important components of the structure/function analysis pipeline. There are several publicly available databases (Table 2) that contain annotations and biochemical RNA secondary structure probing data for analysis of almost any region of the human genome. Many other organisms are well annotated as well (52–53); however, here we will continue to focus on human data. To facilitate their use, we have downloaded select datasets and filtered the annotations (see Methods) to focus on RNA relevant results that we found particularly useful (Data S1). The genome wide data tracks can be uploaded to IGV-ScanFold in their entirety or smaller sections can be extracted (see Loading Annotation and Probing Files) and aligned to the region of interest using genomic coordinates.

**Table 2.** Databases used to generate filtered data for comparison to ScanFold predicted structures

Source	Description	Reference
NCBI	RefSeq Functional Elements	(38)
SNPedia	Single Nucleotide Polymorphisms	(39)
ClinVar	Single Nucleotide Polymorphisms	(40)
TargetScan	Single Nucleotide Polymorphisms	(41)
miRBase	Single Nucleotide Polymorphisms	(42)
ORegAnno	miRNAs and RNA Binding Proteins	(43)
Dfam	Repeat Elements	(44)
RMBase	RNA Modifications	(45)
PolyASite	PolyA sites	(46)
ENCODE	RNA Binding Proteins	(47,48)
RASP	Biochemical Probing Data	(49)

Select databases used to download and generate filtered datasets that can be used to create gene specific annotations or loaded directly into IGV-ScanFold. From left to right: name of the database, description of the data obtained, and reference to each database.

### Application example: the human MYC gene

The *MYC* gene encodes a human transcription factor that is a key component in oncogenesis (54), and has been shown to be dysregulated in over half of all known cancers (55). In previous studies, mature *MYC* mRNA was analyzed using the ScanFold pipeline (56). Here, we utilize IGV-ScanFold predictions across the *MYC* pre-mRNA alongside genome annotations and probing data to provide additional biological context to our previous findings.

### Using IGV-ScanFold on MYC

The most recent version of IGV-ScanFold, was first downloaded from GitHub (https://github.com/ResearchIT/IGV-ScanFold/releases), unzipped and booted using the IGV-ScanFold icon (Mac) or the runme file (Windows). Using the search bar at the top of the window, the gene name or coordinates including the chromosome number (e.g. *MYC* or chr8:127735434−127742951 on the hg38 genome) was entered and the pre-mRNA sequence was loaded. To scan the entire visible region, the ScanFold option at the top of the page was selected followed by ‘Run ScanFold on visible region’. For a more targeted scan of the 3′ UTR, the ‘define region of interest’ button in the menu bar was selected and markers were placed around the region of interest on the cartoon representation of the pre-mRNA, highlighting it red. The red highlighted area was then selected followed by ‘Run ScanFold’. Regardless of the type of scan selected, a new window opened after selecting ‘Run ScanFold’ and the input parameters (i.e., window size, step size, randomizations, shuffle type, temperature, competition, and strand) were adjusted. For this analysis, the default parameters were changed to a 120 nt window size, a 1 nt step size, 100 randomizations per window, mononucleotide shuffling, 37°C temperature, competition of 1 (to demand that only one unique base pair per nucleotide is possible), ‘forward strand’ (using the ‘reverse strand’ option will flip all tracks for genes on the reverse strand), and RNAfold folding algorithm. A detailed discussions of parameters and their optimization can be found in the ScanFold methods paper (13). The default location for IGV-ScanFold output files is in a temporary folder, found using the file path in the parameters log window, but users can also define a permanent directory where all files generated by IGV-ScanFold can be output. After adjusting parameters, selecting the output directory, and clicking ‘Run’, the progress (i.e., estimated time to completion and stage of the run) was monitored in the Log Output window. All output tracks were automatically aligned to the region of interest in the IGV-ScanFold display. A detailed walkthrough of these processes can be found in Video S2.

### Generating and loading annotation and probing files

For many annotation files, it was desirable to extract subsets of the data that only span the region of interest. Despite many processes in IGV that optimize memory use, large annotation and probing data files can still lead to slowdowns. Additionally, when annotation files are pulled from multiple related datasets (e.g., eCLIP and probing data), it may be desirable to compile extracted data into a single, visualizable track for loading into IGV-ScanFold. To facilitate this, we used a python script, FilterBedFiles.py, alongside the annotation files in Data S1 to extract and concatenate annotation and probing data in .bed and .bedgraph file format for the *MYC* pre-mRNA. A demonstration of how this script was used to generate the tracks used in the analysis of the *MYC* pre-mRNA can be found in Video S3. Once annotation and probing tracks were obtained, they were loaded into IGV-ScanFold alongside ScanFold prediction tracks and adjusted according to the user's preference. A detailed walkthrough of how to load and adjust both the view and data displayed on each track can be found in Video S4. All data tracks generated and used in the analysis of *MYC* can be found in Data S5.

### Structural features spanning long transcripts or genomes can be derived from ScanFold-Scan data

**Figure 1.**
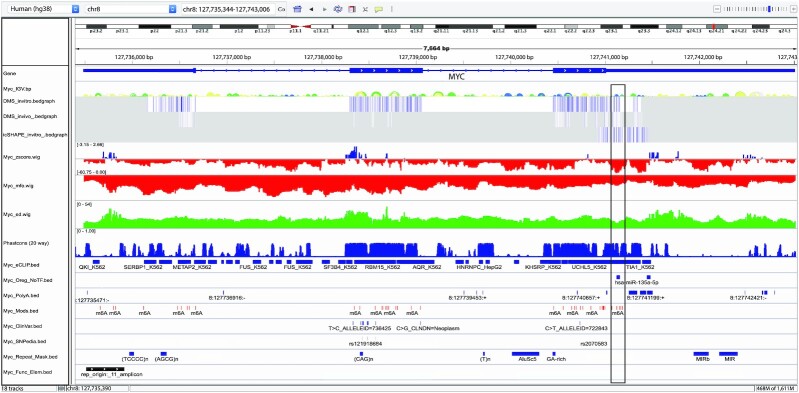
IGV-ScanFold window of all ScanFold, probing, and annotation data for the MYC pre-mRNA. IGV-ScanFold visualization of the entire human *MYC* pre-mRNA. From the top the tracks are: RefSeq genes, ScanFold-Fold base pair track, two DMS probing data sets, one icSHAPE probing data set, ScanFold-Scan*z*-score, ED, and MFE rendered as per nucleotide averages, PhastCons (20-way), eCLIP RBP data, ORegAnno data with no transcription factors, PolyA sites, Refseq functional elements, SNPedia, ClinVar SNPs, repeat elements and RNA modifications. The black box marks the location of Motif 17 (M17) along with all of its metrics, annotations and probing data.

ScanFold-Scan data (i.e. MFE Δ*G*, ED and thermodynamic *z*-score) can be used to derive RNA structural features across the *MYC* pre-mRNA (Figure 1). The thermodynamic stability (predicted MFE ΔG) ranges from −60.75 to 0.00 kcal/mol, which is consistent with previous results on the mature mRNA (56) showing a decrease across the pre-mRNA from the 5′ to 3′ end, as demonstrated by the increasing (less negative/stable) MFE values (Figure 1). This indicates highly stable RNA secondary structure occurs upstream of the *MYC* coding sequence, which may partially explain the presence of an internal ribosomal entry site (IRES) immediately upstream of the *MYC* start codon that allows for bypassing of canonical ribosomal scanning (57,58). Lower thermodynamic stability toward the 3′ UTR could indicate the need to allow for interactions with trans-acting regulatory molecules (e.g. proteins, miRNAs and other non-coding RNAs). Despite thermodynamic stability showing distinct trends across the *MYC* sequence, evidence for ordered thermodynamic stability (measured via the thermodynamic *z*-score); (Figure 1) was observed in distinct regions across the RNA. Overall, *z*-score values ranged from −3.15 to +2.66 and, notably, the most negative peaks (indicative of unusually ordered stability) occurred in the least thermodynamically stable region of *MYC*: its 3′ UTR (Figure 1). Thus, despite being less stable thermodynamically, the structures in the 3′ UTR appear to be specifically ordered to form some of the most stable base pairs possible for that sequence. Similar to the *z*-score, low ED windows appeared across the sequence and frequently overlapped regions of low *z*-score: e.g. in the notable regions of the 3′ UTR (Figure 1). In these regions, predicted ordered stability occurs simultaneously with windows predicted to have low ED values: i.e. the ensemble of conformations is centered on one dominant structure with less evidence for alternative folds.

### Analysis of sequence conservation can indicate functionally significant regions

When sequence conservation, independent of *z*-score: PhastCons scores (measures of unusual nucleotide conservation; (59)) calculated from 20 vertebrate species (Table 2) were overlaid against *MYC* (Figure 1), interesting patterns emerge. As expected, protein coding exonic regions are well conserved, as *MYC* is a key regulatory gene across a variety of species; however, the beginning of the 3′ UTR, that contains exceptionally low *z*-score and ED windows, also showed high levels of sequence conservation. This independently indicates a high likelihood of conserved functionality in this region, thus the constrained evolution of these nucleotides; something that was corroborated in our previous analysis of *MYC* where structural elements in the 3′ UTR were conserved across vertebrates (56). This indicates that some combination of sequence and structural elements in this region of the 3′ UTR appears to be driving its high degree of conservation across many vertebrate species.

### Functional insights can be drawn from analyses of complementary data

Innovations in high-throughput ‘omics’ approaches have resulted in an explosion in the quantity and quality of available data that can be mapped to the genome. Many of these data relate to interactions and effects relevant to RNA structure and biological function (e.g. the data listed in Table 2 and found in Data S5). When these data are aligned with *MYC*, we see that RBP interaction sites (from eCLIP data), microRNA binding sites (predicted and validated), PolyA signals, single nucleotide polymorphisms (SNPs), functional elements, and natural m6A RNA modifications map to sites across the pre-mRNA sequence. The locations of these annotations with respect to ScanFold-Scan and, especially, ScanFold-Fold predictions can facilitate RNA structure/function hypotheses and help determine the goodness of the structure as a potential drug target. An additional type of high-throughput data that has direct relevance to RNA secondary structure are the results from biochemical RNA structure probing data (27). When overlaid versus ScanFold-Scan data, global patterns in reactivity versus stability, *z*-score and ED can be observed. For example, we recently showed that high, or even positive, *z*-score regions correlated with high SHAPE (a single-stranded RNA sensitive reagent) reactivities (13). With robustly predicted secondary structural hypotheses, based on recurring unusually ordered/stable base pairs informed by probing data, and multiple lines of functional evidence overlaid in IGV-ScanFold, it is possible to rapidly home in on regions of exceptional interest.

### Identification of RNA structural motifs with likely functionality and druggability

**Figure 2.**
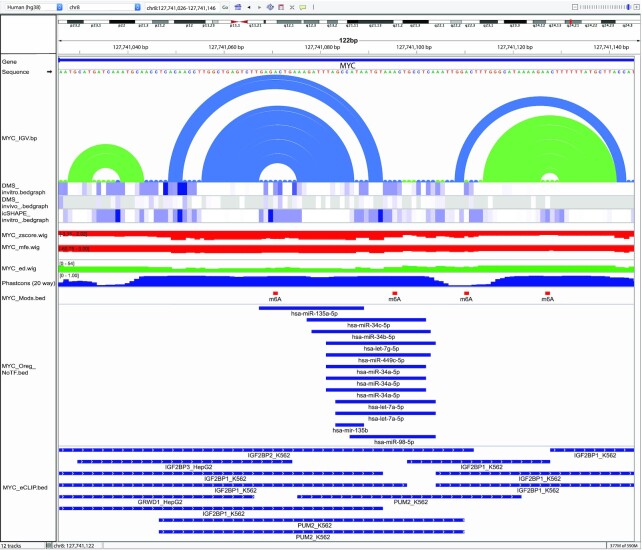
IGV-ScanFold window of all ScanFold, probing, and annotation data for M17 found in the 3′UTR of MYC pre-mRNA. IGV-ScanFold visualization of M17. From the top the tracks are RefSeq genes, sequence, ScanFold-Fold base pair track, two DMS probing data sets, one icSHAPE probing data set, ScanFold-Scan*z*-score, ED and MFE rendered as per nucleotide averages, PhastCons (20-way), RNA modifications, eCLIP RBP data, and ORegAnno data with no transcription factors.

Multiple lines of evidence draw attention to the 3′ UTR of *MYC* (Figure 1): presence of some of the lowest *z*-score and ED windows (−4.38 and 6.95 respectively—both less than 200 nucleotides downstream of the *MYC* stop codon); ScanFold-Fold predicted base pairs with exceptionally low average *z*-scores—notably, supported by in vitro and in vivo biochemical structure probing data (the reactivity of which helps define RNA loops) when they are overlaid versus the ScanFold-Fold model pairs (Figure 1); well conserved nucleotides throughout a variety of vertebrates; as well, multiple functionally significant annotations (e.g. RBP and miRNA binding sites) overlap this region. All lines of evidence point to exceptionally interesting and likely functional RNA structural motifs residing in the 3′ UTR of *MYC*. Indeed, the motif that most stood out was that which we previously discovered: motif (M)17 (56) (Figure 2). Motifs exhibiting these types of metrics can lend themselves as potential drug targets (60,61).

In contrast, to illustrate how one might discard a region unlikely to contain ordered/functional RNA secondary structure, we turn our attention downstream of M17. After ∼bp 127,741,500 (Figure 1) is a cluster of ScanFold-Fold predicted base pairs with negative, but >−1, average *z*-score. These weakly predicted pairs are overlapped by both positive and moderately weak *z*-score prediction windows, making any suggestion of ordered stability ambiguous. The ED is high in this region, indicating a lack of defined RNA folding. Conservation of sequence in this region drops precipitously indicating a lack of any evolutionary constraint on it. This latter observation is corroborated by the lack of any overlapping annotations that would indicate some function for this site. In this case, we would discard the ScanFold predicted motif from further consideration as it is unlikely to have a significant structure/function relationship or form any type of druggable structure.

### 2D structural representations can be generated from ScanFold data

**Figure 3.**
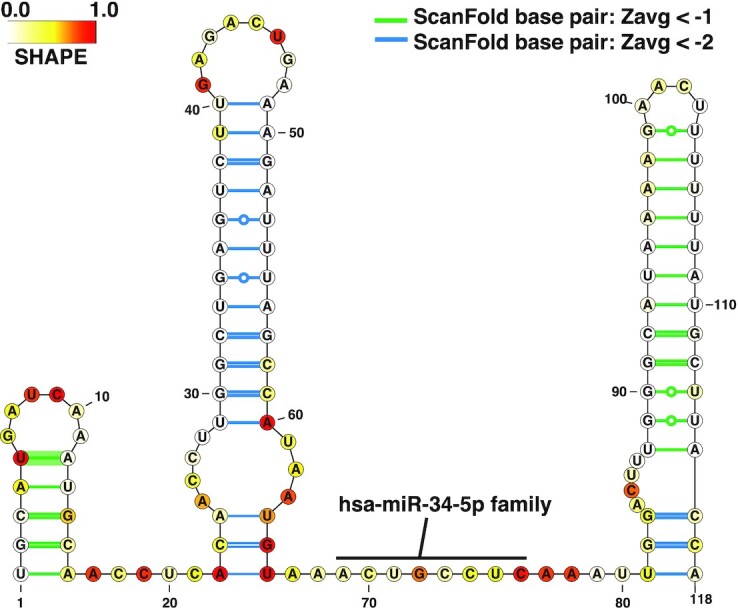
Annotated 2D model of M17 generated using VARNA. A 2D model of M17 created using VARNA. Colors represent *in vivo* icSHAPE biochemical probing reactivity values obtained from the RASP database. White nucleotides represent reactivities of 0, yellow represents reactivities of 0.5, and red represents reactivities of 1.0. Intermediate values are indicated by blended colors. The nucleotide numbering begins at the first base of M17, which corresponds to position 127 741 029 on chr 8.

To aid in the analysis of M17 secondary structure, generating 2D representations was very beneficial. Using the Visualization Applet for RNA (VARNA) program (62) IGV-ScanFold generates basic versions of these models for the entire gene automatically. For a more focused and complete model, the dot bracket files generated from IGV-ScanFold can be readily loaded into VARNA and manipulated. The VARNA 2D model (Figure 3) allows for a more informative and interpretable way to visualize RNA structures of interest alongside additional types of data (e.g. biochemical probing and z-scores) that are available. Within the output directory selected before the scan, all files needed to facilitate structural analyses are found. The files that contain data relevant to generating 2D models are the zscore.wig file (containing average z-scores per nucleotide for heat map generation) and either the ‘Extracted Structures’ or ‘DBNstructures’ files (containing the sequence and dot bracket structures for 2D model generation). For a walkthrough of how to use VARNA to visualize IGV-ScanFold results and complementary data, see Video S6.

Of particular utility, is VARNA’s ability to render biochemical probing data as a heat map: e.g., the in vivo icSHAPE probing (acquired from the RASP database; http://rasp.zhanglab.net/) annotated on Figure 3. The in vivo icSHAPE reactivity is in general agreement with the ScanFold model base pairs; only a short, 3 bp, helix had nucleotides with high icSHAPE reactivity (nucleotides 22−24 and 64−66 in Figure 3). These base pairs are unlikely to form in the cell and, indeed, are consistent with the proposed functional roles of M17 discussed below. It is notable, that the other two available chemical probing datasets also correspond with the ScanFold model of the three hairpins in M17 (Figure 2).

### Generating structure/function hypotheses with ScanFold

With all discussed data tracks overlaid, one of the most remarkable results for *MYC* is the presence of multiple miRNA binding sites on M17 (Figure 2); this is particularly striking when these sites are overlaid on the 2D model (Figure 3). Significantly, miRNA seed binding occurs in a single-stranded stretch of M17 between two highly ordered hairpins; where additional intermolecular base pairs are possible with the highly SHAPE-reactive nucleotides that comprise the 3 base pair helix. Interestingly, our previous analyses of M17 found mutations that extend base pairing and stabilize the predicted helix (‘zipping up’ the internal loop) ablated miRNA mediated gene regulation in a dual luciferase reporter system, while mutations that destabilize the helix had no effect (56). Thus, a presumable function of M17 is to control accessibility of miRNA binding sites, potentially via the interplay of RNA structure and various binding proteins.

Additional evidence for post-transcriptional regulatory roles of M17 comes from the analysis of RBP binding sites identified from recently available eCLIP data (47,48) (Figure 2). Notable RBPs include the translational repressor and cell proliferation regulator PUM2 and the translational and microRNA-mediated degradation regulators IGF2BP1-3; which could suggest that M17 is involved in regulation of gene expression, cell viability, and localization (63–65) via modulation of interactions with these regulatory RBPs. Interestingly, four m6A modifications also overlap the RBP interaction sites on M17 (Figure 2). Two of these modifications occur within the single-stranded stretch containing miRNA interaction sites (one falling within seed interaction sites), while the other two occur within the terminal loops of the flanking hairpins. These modifications could thus, potentially, be modulating interactions with both RBPs and miRNAs (66,67)

## CONCLUSION

IGV-ScanFold serves as a tool for fast and effective functional RNA structure discovery. With the increased capabilities of IGV-ScanFold, functional secondary structure can be predicted, visualized, and analyzed against annotation and probing data quickly and easily. With surging interest in RNA biology (e.g. due the central role played by RNA in both causing and treating the COVID-19 pandemic) and advancements being made in using RNA as a therapeutic agent and target, this new tool will be of great utility. Specifically, by adding RNA functional analysis and structural motif discovery capabilities to a widely used tool, IGV, we hope that a wide array of researchers will be able to easily incorporate such analyses into their pre-existing discovery pipelines. As additional omics-scale datasets become available and innovations unlock new areas of RNA biology, having an efficient tool for integrating and analyzing such diverse information will help drive new discoveries.

## DATA AVAILABILITY

All Datasets and Videos (S1-S6) associated with this study are available at: https://structurome.bb.iastate.edu/igv-scanfold-supplemental-data.

All supplemental videos are also available at https://www.youtube.com/playlist?list=PL-FLz2olO5ubN8hKbeccZ6rfwBjfmTSyv.

The IGV-ScanFold bundle releases are available at: https://github.com/ResearchIT/IGV-ScanFold/releases.

## SUPPLEMENTARY DATA

Supplementary Data are available at NARGAB Online.

## References

[1] Bernat V. , Disney M.D. RNA structures as mediators of neurological diseases and as drug targets. Neuron. 2015; 87:28–46. 26139368 10.1016/j.neuron.2015.06.012 PMC4508199

[2] Fabbri M. , Girnita L., Varani G., Calin G.A. Decrypting noncoding RNA interactions, structures, and functional networks. Genome Res. 2019; 29:1377–1388. 31434680 10.1101/gr.247239.118 PMC6724670

[3] Mercer T.R. , Mattick J.S. Structure and function of long noncoding RNAs in epigenetic regulation. Nat. Struct. Mol. Biol. 2013; 20:300–307. 23463315 10.1038/nsmb.2480

[4] Mortimer S.A. , Kidwell M.A., Doudna J.A. Insights into RNA structure and function from genome-wide studies. Nat. Rev. Genet. 2014; 15:469–479. 24821474 10.1038/nrg3681

[5] Jiang P. , Coller H. Functional interactions between microRNAs and RNA binding proteins. Microrna. 2012; 1:70–79. 25048093 10.2174/2211536611201010070 PMC5123774

[6] Mauger D.M. , Cabral B.J., Presnyak V., Su S.V., Reid D.W., Goodman B., Link K., Khatwani N., Reynders J., Moore M.J. et al. mRNA structure regulates protein expression through changes in functional half-life. Proc. Natl. Acad. Sci. U.S.A. 2019; 116:24075–24083. 31712433 10.1073/pnas.1908052116 PMC6883848

[7] Shi B. , Zhang J., Heng J., Gong J., Zhang T., Li P., Sun B.F., Yang Y., Zhang N., Zhao Y.L. et al. RNA structural dynamics regulate early embryogenesis through controlling transcriptome fate and function. Genome Biol. 2020; 21:120 32423473 10.1186/s13059-020-02022-2 PMC7236375

[8] Gruber A.R. , Findeiss S., Washietl S., Hofacker I.L., Stadler P.F. RNAz 2.0: improved noncoding RNA detection. Pac. Symp. Biocomput. 2010; 1:69–79. 19908359

[9] Pedersen J.S. , Bejerano G., Siepel A., Rosenbloom K., Lindblad-Toh K., Lander E.S., Kent J., Miller W., Haussler D Identification and classification of conserved RNA secondary structures in the human genome. PLoS Comput. Biol. 2006; 2:e33 16628248 10.1371/journal.pcbi.0020033 PMC1440920

[10] Yao Z. , Weinberg Z., Ruzzo W.L. CMfinder–a covariance model based RNA motif finding algorithm. Bioinformatics. 2006; 22:445–452. 16357030 10.1093/bioinformatics/btk008

[11] Heyne S. , Costa F., Rose D., Backofen R. GraphClust: alignment-free structural clustering of local RNA secondary structures. Bioinformatics. 2012; 28:i224–i232. 22689765 10.1093/bioinformatics/bts224 PMC3371856

[12] Gutell R.R. , Power A., Hertz G.Z., Putz E.J., Stormo G.D. Identifying constraints on the higher-order structure of RNA: continued development and application of comparative sequence analysis methods. Nucleic Acids Res. 1992; 20:5785–5795. 1454539 10.1093/nar/20.21.5785 PMC334417

[13] Andrews R.J. , Baber L., Moss W.N. Mapping the RNA structural landscape of viral genomes. Methods. 2020; 183:57–67. 31711930 10.1016/j.ymeth.2019.11.001 PMC7205576

[14] Gardner P.P. , Giegerich R. A comprehensive comparison of comparative RNA structure prediction approaches. BMC Bioinformatics. 2004; 5:140 15458580 10.1186/1471-2105-5-140 PMC526219

[15] Andrews R.J. , Roche J., Moss W.N. ScanFold: an approach for genome-wide discovery of local RNA structural elements-applications to Zika virus and HIV. PeerJ. 2018; 6:e6136 30627482 10.7717/peerj.6136 PMC6317755

[16] Lorenz R. , Bernhart S.H., Honer Zu Siederdissen C., Tafer H., Flamm C., Stadler P.F., Hofacker I.L. ViennaRNA Package 2.0. Algorithms Mol. Biol. 2011; 6:26 22115189 10.1186/1748-7188-6-26 PMC3319429

[17] Andrews R.J. , O’Leary C.A., Tompkins V.S., Peterson J.M., Haniff H.S., Williams C., Disney M.D., Moss W.N. A map of the SARS-CoV-2 RNA structurome. NAR. Genom. Bioinform. 2021; 3:lqab043 34046592 10.1093/nargab/lqab043 PMC8140738

[18] Manfredonia I. , Nithin C., Ponce-Salvatierra A., Ghosh P., Wirecki T.K., Marinus T., Ogando N.S., Snijder E.J., van Hemert M.J., Bujnicki J.M. et al. Genome-wide mapping of SARS-CoV-2 RNA structures identifies therapeutically-relevant elements. Nucleic Acids Res. 2020; 48:12436–12452. 33166999 10.1093/nar/gkaa1053 PMC7736786

[19] Nawrocki E.P. , Eddy S.R. Infernal 1.1: 100-fold faster RNA homology searches. Bioinformatics. 2013; 29:2933–2935. 24008419 10.1093/bioinformatics/btt509 PMC3810854

[20] Rivas E. , Clements J., Eddy S.R. A statistical test for conserved RNA structure shows lack of evidence for structure in lncRNAs. Nat. Methods. 2017; 14:45–48. 27819659 10.1038/nmeth.4066 PMC5554622

[21] Clote P. , Ferre F., Kranakis E., Krizanc D Structural RNA has lower folding energy than random RNA of the same dinucleotide frequency. RNA. 2005; 11:578–591. 15840812 10.1261/rna.7220505 PMC1370746

[22] Freyhult E. , Gardner P.P., Moulton V. A comparison of RNA folding measures. BMC Bioinformatics. 2005; 6:241 16202126 10.1186/1471-2105-6-241 PMC1274297

[23] Su C. , Weir J.D., Zhang F., Yan H., Wu T. ENTRNA: a framework to predict RNA foldability. BMC Bioinformatics. 2019; 20:373 31269893 10.1186/s12859-019-2948-5 PMC6610807

[24] Moss W.N. The ensemble diversity of non-coding RNA structure is lower than random sequence. Noncoding RNA Res. 2018; 3:100–107. 30175283 10.1016/j.ncrna.2018.04.005 PMC6114264

[25] Busan S. , Weeks K.M. Visualization of RNA structure models within the Integrative Genomics Viewer. RNA. 2017; 23:1012–1018. 28428329 10.1261/rna.060194.116 PMC5473135

[26] Haddad C. , Davila-Calderon J., Tolbert B.S. Integrated approaches to reveal mechanisms by which RNA viruses reprogram the cellular environment. Methods. 2020; 183:50–56. 32622045 10.1016/j.ymeth.2020.06.013 PMC7329689

[27] Mitchell D. 3rd, Assmann S.M., Bevilacqua P.C. Probing RNA structure in vivo. Curr. Opin. Struct. Biol. 2019; 59:151–158. 31521910 10.1016/j.sbi.2019.07.008 PMC6888943

[28] Rangan R. , Zheludev I.N., Hagey R.J., Pham E.A., Wayment-Steele H.K., Glenn J.S., Das R. RNA genome conservation and secondary structure in SARS-CoV-2 and SARS-related viruses: a first look. RNA. 2020; 26:937–959. 32398273 10.1261/rna.076141.120 PMC7373990

[29] Zhang P. , Park H.J., Zhang J., Junn E., Andrews R.J., Velagapudi S.P., Abegg D., Vishnu K., Costales M.G., Childs-Disney J.L. et al. Translation of the intrinsically disordered protein alpha-synuclein is inhibited by a small molecule targeting its structured mRNA. Proc. Natl. Acad. Sci. U.S.A. 2020; 117:1457–1467. 31900363 10.1073/pnas.1905057117 PMC6983430

[30] Benhamou R.I. , Angelbello A.J., Andrews R.J., Wang E.T., Moss W.N., Disney M.D. Structure-specific cleavage of an RNA repeat expansion with a dimeric small molecule is advantageous over sequence-specific recognition by an oligonucleotide. ACS Chem. Biol. 2020; 15:485–493. 31927948 10.1021/acschembio.9b00958 PMC7081929

[31] Tomezsko P.J. , Corbin V.D.A., Gupta P., Swaminathan H., Glasgow M., Persad S., Edwards M.D., McIntosh L., Papenfuss A.T., Emery A. et al. Determination of RNA structural diversity and its role in HIV-1 RNA splicing. Nature. 2020; 582:438–442. 32555469 10.1038/s41586-020-2253-5 PMC7310298

[32] Morandi E. , Manfredonia I., Simon L.M., Anselmi F., van Hemert M.J., Oliviero S., Incarnato D. Genome-scale deconvolution of RNA structure ensembles. Nat. Methods. 2021; 18:249–252. 33619392 10.1038/s41592-021-01075-w

[33] Haniff H.S. , Tong Y., Liu X., Chen J.L., Suresh B.M., Andrews R.J., Peterson J.M., O’Leary C.A., Benhamou R.I., Moss W.N. et al. Targeting the SARS-CoV-2 RNA genome with small molecule binders and ribonuclease targeting chimera (RIBOTAC) degraders. ACS Cent. Sci. 2020; 6:1713–1721. 33140033 10.1021/acscentsci.0c00984 PMC7553039

[34] Andrews R.J. , Moss W.N. Computational approaches for the discovery of splicing regulatory RNA structures. Biochim. Biophys. Acta Gene. Regul. Mech. 2019; 1862:194380 31048028 10.1016/j.bbagrm.2019.04.007 PMC6819252

[35] Robinson J.T. , Thorvaldsdottir H., Winckler W., Guttman M., Lander E.S., Getz G., Mesirov J.P. Integrative genomics viewer. Nat. Biotechnol. 2011; 29:24–26. 21221095 10.1038/nbt.1754 PMC3346182

[36] Thorvaldsdottir H. , Robinson J.T., Mesirov J.P. Integrative Genomics Viewer (IGV): high-performance genomics data visualization and exploration. Brief Bioinform. 2013; 14:178–192. 22517427 10.1093/bib/bbs017 PMC3603213

[37] Andrews R.J. , Baber L., Moss W.N. RNAStructuromeDB: a genome-wide database for RNA structural inference. Sci. Rep. 2017; 7:17269 29222504 10.1038/s41598-017-17510-y PMC5722888

[38] O'Leary N.A. , Wright M.W., Brister J.R., Ciufo S., Haddad D., McVeigh R., Rajput B., Robbertse B., Smith-White B., Ako-Adjei D. et al. Reference sequence (RefSeq) database at NCBI: current status, taxonomic expansion, and functional annotation. Nucleic Acids Res. 2016; 44:D733–D745. 26553804 10.1093/nar/gkv1189 PMC4702849

[39] Cariaso M. , Lennon G. SNPedia: a wiki supporting personal genome annotation, interpretation and analysis. Nucleic Acids Res. 2012; 40:D1308–D1312. 22140107 10.1093/nar/gkr798 PMC3245045

[40] Landrum M.J. , Lee J.M., Riley G.R., Jang W., Rubinstein W.S., Church D.M., Maglott D.R. ClinVar: public archive of relationships among sequence variation and human phenotype. Nucleic Acids Res. 2014; 42:D980–D985. 24234437 10.1093/nar/gkt1113 PMC3965032

[41] Agarwal V. , Bell G.W., Nam J.W., Bartel D.P. Predicting effective microRNA target sites in mammalian mRNAs. Elife. 2015; 4:e05005 10.7554/eLife.05005 PMC4532895 26267216

[42] Kozomara A. , Birgaoanu M., Griffiths-Jones S. miRBase: from microRNA sequences to function. Nucleic Acids Res. 2019; 47:D155–D162. 30423142 10.1093/nar/gky1141 PMC6323917

[43] Lesurf R. , Cotto K.C., Wang G., Griffith M., Kasaian K., Jones S.J., Montgomery S.B. ORegAnno 3.0: a community-driven resource for curated regulatory annotation. Nucleic Acids Res. 2016; 44:D126–D132. 26578589 10.1093/nar/gkv1203 PMC4702855

[44] Hubley R. , Finn R.D., Clements J., Eddy S.R., Jones T.A., Bao W., Smit A.F., Wheeler T.J. The Dfam database of repetitive DNA families. Nucleic Acids Res. 2016; 44:D81–D89. 26612867 10.1093/nar/gkv1272 PMC4702899

[45] Xuan J.J. , Sun W.J., Lin P.H., Zhou K.R., Liu S., Zheng L.L., Qu L.H., Yang J.H. RMBase v2.0: deciphering the map of RNA modifications from epitranscriptome sequencing data. Nucleic Acids Res. 2018; 46:D327–D334. 29040692 10.1093/nar/gkx934 PMC5753293

[46] Herrmann C.J. , Schmidt R., Kanitz A., Artimo P., Gruber A.J., Zavolan M. PolyASite 2.0: a consolidated atlas of polyadenylation sites from 3' end sequencing. Nucleic Acids Res. 2020; 48:D174–D179. 31617559 10.1093/nar/gkz918 PMC7145510

[47] Davis C.A. , Hitz B.C., Sloan C.A., Chan E.T., Davidson J.M., Gabdank I., Hilton J.A., Jain K., Baymuradov U.K., Narayanan A.K. et al. The Encyclopedia of DNA elements (ENCODE): data portal update. Nucleic Acids Res. 2018; 46:D794–D801. 29126249 10.1093/nar/gkx1081 PMC5753278

[48] Van Nostrand E.L. , Freese P., Pratt G.A., Wang X., Wei X., Xiao R., Blue S.M., Chen J.Y., Cody N.A.L., Dominguez D. et al. A large-scale binding and functional map of human RNA-binding proteins. Nature. 2020; 583:711–719. 32728246 10.1038/s41586-020-2077-3 PMC7410833

[49] Li P. , Zhou X., Xu K., Zhang Q.C. RASP: an atlas of transcriptome-wide RNA secondary structure probing data. Nucleic Acids Res. 2021; 49:D183–D191. 33068412 10.1093/nar/gkaa880 PMC7779053

[50] Kuhn R.M. , Haussler D., Kent W.J. The UCSC genome browser and associated tools. Brief Bioinform. 2013; 14:144–161. 22908213 10.1093/bib/bbs038 PMC3603215

[51] Reuter J.S. , Mathews D.H. RNAstructure: software for RNA secondary structure prediction and analysis. BMC Bioinformatics. 2010; 11:129 20230624 10.1186/1471-2105-11-129 PMC2984261

[52] Alliance of Genome Resources C. Alliance of Genome Resources Portal: unified model organism research platform. Nucleic Acids Res. 2020; 48:D650–D658. 31552413 10.1093/nar/gkz813 PMC6943066

[53] Haeussler M. , Zweig A.S., Tyner C., Speir M.L., Rosenbloom K.R., Raney B.J., Lee C.M., Lee B.T., Hinrichs A.S., Gonzalez J.N. et al. The UCSC Genome Browser database: 2019 update. Nucleic Acids Res. 2019; 47:D853–D858. 30407534 10.1093/nar/gky1095 PMC6323953

[54] Dang C.V. MYC on the path to cancer. Cell. 2012; 149:22–35. 22464321 10.1016/j.cell.2012.03.003 PMC3345192

[55] Dang C.V. , Reddy E.P., Shokat K.M., Soucek L. Drugging the ‘undruggable’ cancer targets. Nat. Rev. Cancer. 2017; 17:502–508. 28643779 10.1038/nrc.2017.36 PMC5945194

[56] O’Leary C.A. , Andrews R.J., Tompkins V.S., Chen J.L., Childs-Disney J.L., Disney M.D., Moss W.N. RNA structural analysis of the MYC mRNA reveals conserved motifs that affect gene expression. PLoS One. 2019; 14:e0213758 31206539 10.1371/journal.pone.0213758 PMC6576772

[57] Stoneley M. , Subkhankulova T., Le Quesne J.P., Coldwell M.J., Jopling C.L., Belsham G.J., Willis A.E. Analysis of the c-myc IRES; a potential role for cell-type specific trans-acting factors and the nuclear compartment. Nucleic Acids Res. 2000; 28:687–694. 10637319 10.1093/nar/28.3.687 PMC102558

[58] Le Quesne J.P. , Stoneley M., Fraser G.A., Willis A.E. Derivation of a structural model for the c-myc IRES. J. Mol. Biol. 2001; 310:111–126. 11419940 10.1006/jmbi.2001.4745

[59] Siepel A. , Bejerano G., Pedersen J.S., Hinrichs A.S., Hou M., Rosenbloom K., Clawson H., Spieth J., Hillier L.W., Richards S. et al. Evolutionarily conserved elements in vertebrate, insect, worm, and yeast genomes. Genome Res. 2005; 15:1034–1050. 16024819 10.1101/gr.3715005 PMC1182216

[60] Falese J.P. , Donlic A., Hargrove A.E. Targeting RNA with small molecules: from fundamental principles towards the clinic. Chem. Soc. Rev. 2021; 50:2224–2243. 33458725 10.1039/d0cs01261k PMC8018613

[61] Donlic A. , Hargrove A.E. Targeting RNA in mammalian systems with small molecules. Wiley Interdiscip. Rev. RNA. 2018; 9:e1477 29726113 10.1002/wrna.1477 PMC6002909

[62] Darty K. , Denise A., Ponty Y. VARNA: Interactive drawing and editing of the RNA secondary structure. Bioinformatics. 2009; 25:1974–1975. 19398448 10.1093/bioinformatics/btp250 PMC2712331

[63] Smialek M.J. , Ilaslan E., Sajek M.P., Jaruzelska J. Role of PUM RNA-binding proteins in cancer. Cancers (Basel.). 2021; 13:129–142. 10.3390/cancers13010129 PMC7796173 33401540

[64] Wachter K. , Kohn M., Stohr N., Huttelmaier S. Subcellular localization and RNP formation of IGF2BPs (IGF2 mRNA-binding proteins) is modulated by distinct RNA-binding domains. Biol. Chem. 2013; 394:1077–1090. 23640942 10.1515/hsz-2013-0111

[65] Lambrianidou A. , Sereti E., Soupsana K., Komini C., Dimas K., Trangas T. mTORC2 deploys the mRNA binding protein IGF2BP1 to regulate c-MYC expression and promote cell survival. Cell Signal. 2021; 80:109912 33388443 10.1016/j.cellsig.2020.109912

[66] Zhou Y. , Kong Y., Fan W., Tao T., Xiao Q., Li N., Zhu X. Principles of RNA methylation and their implications for biology and medicine. Biomed. Pharmacother. 2020; 131:110731 32920520 10.1016/j.biopha.2020.110731

[67] Huang H. , Weng H., Chen J. m(6)A modification in coding and non-coding RNAs: roles and therapeutic implications in cancer. Cancer Cell. 2020; 37:270–288. 32183948 10.1016/j.ccell.2020.02.004 PMC7141420

